# National Survey on Implementation Patterns and Functional Needs for Hospital-Wide Glycemic Management Systems in the Chinese Mainland: Mixed Methods Study

**DOI:** 10.2196/90796

**Published:** 2026-08-28

**Authors:** Xiazi Wang, Jin Huang, Tianhui Xu, Zhe Zhang, Chenshuang Luo, Yaqiong Tan, Huiping Wang, Rong Xu

**Affiliations:** 1 Clinical Nursing Teaching and Research Section the Second Xiangya Hospital Central South University Changsha, Hunan China; 2 National Clinical Research Center for Endocrine and Metabolic Diseases Changsha, Hunan China; 3 Department of Metabolism and Endocrinology the Second Xiangya Hospital Central South University Changsha, Hunan China; 4 Xiangya School of Nursing Central South University Changsha, Hunan China

**Keywords:** diabetes mellitus, hospital-wide glycemic management, hospital-wide glycemic management systems, HGMS, health care informatization, multidisciplinary collaboration, mixed methods study

## Abstract

**Background:**

Diabetes mellitus is a major global public health burden, and achieving optimal glycemic control remains challenging. Multidisciplinary, hospital-wide glycemic management systems (HGMS) have emerged as a promising strategy. In the Chinese mainland, the adoption of HGMS has accelerated; yet, substantial variations remain in system functionality, regional coverage, and management effectiveness across hospitals, underscoring the need for a national survey.

**Objective:**

This study aimed to survey the implementation patterns and functional needs for HGMS in the Chinese mainland.

**Methods:**

A mixed methods design was used, integrating a cross-sectional quantitative survey and qualitative interviews. From April 2024 to December 2024, health care professionals across 31 provincial-level administrative regions in the Chinese mainland were recruited. Data were collected using standardized questionnaires and purposive semistructured interviews to evaluate the adoption, functional demands, and satisfaction with HGMS. Quantitative data were analyzed using SPSS (version 27.0; IBM Corp). Differences in mean ratings across HGMS functions were evaluated using the Friedman test, with post hoc pairwise comparisons performed via the Wilcoxon signed-rank test and Bonferroni correction. Qualitative data underwent thematic analysis following Braun and Clarke’s framework, supported by NVivo (version 12; QSR International).

**Results:**

A total of 988 health care professionals from 265 hospitals participated. HGMS implementation across hospitals in the Chinese mainland exhibited pronounced heterogeneity: 12.45% remained at level 0 (handwritten glucose records), 47.92% at level 1 (manual data entry), and 21.51% at level 2 (department-level data sharing). Levels 3, 4, and 5 accounted for 13.21%, 3.40%, and 1.51%, respectively, revealing significant regional disparities. Functional demand analysis indicated consensus on the importance of basic HGMS functions, including real-time hypo- or hyperglycemia alerts (68.42%), Computerized Physician Order Entry–Electronic Medical Record interoperability (68.02%), and automatic glucose data transmission (67.51%). Among advanced functions, standardized education and training (64.47%), clinical decision support (61.54%), and tele-endocrinology consultation (61.13%) were also valued. Satisfaction was highest for automatic glucose data transmission (81.63%), while clinical decision support received the lowest satisfaction (45.51%). The Friedman test indicated significant differences in perceived importance and satisfaction across the 7 functions (*χ*²_6_=89.895, 286.680, respectively; *P*<.001). Post hoc analyses with Bonferroni adjustment revealed that participants ranked the relative importance and satisfaction of functions in distinct orders, with automated glucose data transmission consistently rated highest for both. Qualitative analysis revealed persistent barriers in nonspecialist wards, including insufficient information integration and collaboration, absence of risk stratification and misaligned management, and insufficient knowledge and education need.

**Conclusions:**

HGMS implementation in the Chinese mainland is generally transitioning from digitized glucose recording toward intelligent glycemic management across hospitals. Future efforts should focus on establishing virtual wards for integrated care, developing dynamic glucose monitoring-guided management pathways, creating patient-centered collaborative platforms, and incorporating AI-enhanced clinical decision support to overcome existing barriers.

## Introduction

Diabetes mellitus is a metabolic disorder characterized by impaired insulin secretion and/or insulin action, leading to persistent hyperglycemia as its hallmark feature [1]. Driven by population aging, dietary transitions, and sedentary lifestyles, the global prevalence of diabetes has risen sharply, posing a major challenge to public health systems [2]. This growing burden is evident in global surveillance data. According to the 11th edition of the International Diabetes Federation Diabetes Atlas, an estimated 589 million adults aged 20-79 years were living with diabetes worldwide in 2024. This number is projected to rise to 853 million by 2050 [3]. The disease burden is particularly concentrated in developing countries, with China (148 million cases) ranking first globally, followed by India (89.8 million) and the United States (38.5 million) [3,4]. Diabetes is among the leading causes of mortality worldwide, responsible for over 3.4 million deaths in 2024, accounting for 9.3% of all-cause mortality [3]. Therefore, effective monitoring and control of blood glucose levels are essential to reduce complications and improve patient outcomes.

Optimal glycemic control plays a critical role in delaying disease progression and preventing complications [5]. Evidence shows that among hospitalized patients outside endocrinology wards, approximately 30% experience hyperglycemia during admission, with 26% having a prior diagnosis of diabetes [6]. Poor glycemic control increases the risk of wound infection and other complications, prolongs hospital stays, and severe hypoglycemia may even be life-threatening [7]. Traditionally, inpatient glycemic management has relied on department-specific monitoring or consultations with endocrinology specialists [8]. Although these approaches can partially improve glycemic target achievement, they remain insufficient for hospital-wide management. In 2012, the Endocrine Society in the United States recommended establishing standardized, evidence-based inpatient glycemic management systems [9]. However, conventional approaches still face substantial limitations, including inconsistent standards, delayed monitoring, and untimely interventions, which hinder their capacity to meet hospital-wide needs. These challenges underscore the need for digital health innovations to enable more systematic, standardized, and efficient glucose management.

Building on this digital transformation trend, hospital-wide glycemic management systems (HGMS) have emerged as a key strategy [10]. HGMS are typically led by endocrinology departments and rely on digital platforms interoperable with hospital information systems [11,12]. This integration enables timely remote management by endocrinology specialists, including tailored glucose education, monitoring, and individualized therapeutic adjustments [13,14]. In countries such as the United States and the United Kingdom, such digital systems have enabled real-time glucose tracking, automated alerts, and AI-assisted insulin titration, resulting in improved safety and shorter hospital stays [15-17]. However, the implementation of HGMS in the Chinese mainland remains heterogeneous, with substantial disparities in infrastructure and clinical usage across hospitals [18,19]. Existing research is largely limited to small-scale pilot projects, lacking national-level data on adoption, functionality, and user experience [20]. This absence of large-scale evidence restricts informed decision-making at both hospital and policy levels. Therefore, a nationwide, systematic survey is essential to comprehensively evaluate the application of the systems across different regions and hospital tiers. By generating nationally representative data, this study can inform hospitals in optimizing resource allocation, refining management processes, and advancing equitable approaches to glycemic management.

## Methods

### Study Design

We used an explanatory sequential mixed methods design, comprising a nationwide, multicenter online cross-sectional survey followed by semistructured interviews. The quantitative phase generated a comprehensive overview of HGMS adoption and usage patterns across hospitals, and findings from this phase informed purposive sampling and the topic guide for the subsequent qualitative phase. The qualitative interviews further explored contextual factors, facilitators, and barriers, thereby enabling a richer interpretation of observed quantitative patterns.

### Setting

The survey was developed and managed by the National Clinical Research Center for Metabolic Diseases and administered via the professional online survey platform Sojump (Changsha Ranxing Information Technology Co, Ltd). Data collection occurred over a 9-month period (April-December 2024). The platform allowed secure data export and IP logging. The survey invitation included (1) study background and objectives, (2) an informed consent statement and voluntary participation declaration, (3) assurances of confidentiality and anonymity, and (4) a link to the questionnaire. Responses were collected anonymously; all items were set as required to minimize partial records, and submissions were limited to 1 per IP address.

### Recruitment

Participants were recruited nationwide through internal hospital announcements and professional academic platforms to maximize geographic and hospitals coverage. Inclusion criteria were (1) possession of a valid nursing or medical practitioner license with at least 1 year of clinical experience; (2) involvement in the oversight, coordination, implementation, or quality management of glucose management; and (3) voluntary participation and provision of signed informed consent. Exclusion criteria were (1) nonclinical personnel, such as logistics staff; and (2) individuals on long-term leave during the survey period. A total of 988 clinical health care professionals from 265 hospitals across the Chinese mainland participated in the study, covering hospitals at all levels and key departments such as internal medicine, surgery, intensive care units, and emergency departments. For the qualitative research, we used purposive sampling to select interview participants from the survey respondents. They were identified and approached by telephone, and a total of 15 individuals consented to take part.

### Measurement and Data Collection

The survey questionnaire was developed using an expert consensus approach. The expert panel comprised 3 endocrinologists, 2 nursing scholars, and 1 diabetes nurse educator. Based on current blood glucose management guidelines and clinical needs, the panel designed the questionnaire items. All questions were reviewed through expert consultation and pilot testing to ensure the scientific rigor and reliability of the instrument.

The questionnaire consisted of four sections:

1. Demographic information: Multiple-choice and fill-in-the-blank questions were used to collect participants’ basic information, including gender, age, years of work experience, and so on.
2. Current status of HGMS levels: Multiple-choice questions assessed the levels of glycemic management systems adopted by the respondents’ hospitals, covering 6 levels defined by expert consensus; the details are shown in Table 1. This consensus was developed through a structured process incorporating 3 core dimensions (glucometer types, informatization level, and departmental coverage), weighted scoring, and the Delphi method, representing a credible and practical standard for HGMS levels [21].
3. Perceived importance of HGMS’ functions: Participants rated the importance of core system functions (1=very unimportant to 5=very important), which were categorized into basic functions (automated glucose data transmission, real-time hypo- or hyperglycemia alerts, and Computerized Physician Order Entry–Electronic Medical Record interoperability) and advanced functions (tele-endocrinology consultation, standardized education and training, clinical decision support, and multidisciplinary remote glucose management).
4. Satisfaction with HGMS’ functions: Participants were invited to report their perceived satisfaction by 5-point Likert scales (1=very dissatisfied to 5=very satisfied) with the system, including data accuracy, alert responsiveness, system integration, and usability.

For qualitative data, we conducted a semistructured interview (Textbox 1). Data collection continued until thematic saturation was achieved, as determined by 3 consecutive interviews yielding no new interdisciplinary theme. All questions were open-ended, and each interview lasted approximately 20~30 minutes.

**Table 1:** Levels of glycemic management systems.

Levels		Definition
**Level 0**		
	No information system	Handwritten blood glucose records only.
**Level 1**		
	Preliminary informatization	Manual entry of glucose records into an electronic system without cross-departmental data exchange.
**Level 2**		
	Departmental data sharing	Sharing establishes information systems where data are shared and processed within departments. And smart glucometers used in some departments enable automatic data transmission and integration with the hospital information system, triggering automatic alerts when patients exceed relevant target ranges.
**Level 3**		
	Hospital-wide data sharing	Hospital-wide adoption enabling data exchange and authorized cross-departmental access to glucose data.
**Level 4**		
	Regional data sharing	Hospitals within the region adopt information-based management, enabling regional data sharing. Enables data exchange between major systems via data interfaces, featuring business data verification functionality.
**Level 5**		
	Home-based smart management	Patients use home glucometers with automatic data transmission enabling remote clinician monitoring and decision support, such as automatic indicator generation and integrated information display.

**Interview outline.**

- In your opinion, how effective is current inpatient glycemic management in nonendocrinology department at your institution?
- What would you identify as the most common or prominent challenges in hospital-wide glycemic management initiatives?
- What are the most frequent cognitive or practical misconceptions held by colleagues in nonspecialist departments regarding routine glycemic management?
- Could you describe a specific case that illustrates how such misconceptions or difficulties impact clinical practice, and how they were ultimately resolved?
- When encountering challenges in glycemic management, what forms of immediate support from the endocrinology team do you find most valuable?
- From a long-term perspective, in what ways can the endocrinology team enhance the capacity and confidence of nonspecialist medical staff in autonomous glycemic management?
- What key support does the hospital-wide glycemic management system provide in addressing glycemic control challenges?
- Based on your experience, which aspects of the hospital-wide glycemic management system could be optimized, and are there any unmet clinical needs?

### Data Analysis

Quantitative data were analyzed by SPSS (version 27.0; IBM Corp). Categorical variables were summarized as frequencies (n), corresponding percentages, and mean (SD) to provide a comprehensive overview of the distribution of HGMS usage in the Chinese mainland. Given that the distributions of importance and satisfaction ratings severely deviated from normality, nonparametric alternatives were applied. Differences in mean ratings of importance and satisfaction across the 7 HGMS functions were analyzed using the Friedman test. Post hoc pairwise comparisons were conducted using the Wilcoxon signed-rank test with Bonferroni correction to identify significant differences between individual functions. Statistical significance was set at *P*<.05.

Qualitative data were analyzed using Braun and Clarke’s thematic analysis [22]. All interviews were audio-recorded, transcribed verbatim, and imported into NVivo (version 14) for data management and analysis. The research team began by repeatedly reading the transcripts to gain familiarity with the data, after which the texts were segmented into meaningful units and assigned initial codes. Through constant comparison, conceptually related codes were grouped into categories and further consolidated into overarching themes. Iterative analysis continued until there was no new interdisciplinary theme, indicating theoretical saturation. Two researchers (XW and TX) independently conducted the coding and theme refinement. To ensure coding reliability, they first independently coded the initial 5 transcripts and met to resolve discrepancies through discussion. The remaining transcripts were coded independently, with regular meetings scheduled to discuss any emerging disagreements. Any discrepancies were resolved through consultation with a third qualitative expert (RX). Repeated checking and comparison throughout the process ensured internal coherence among the codes, categories, and final themes.

### Bias Control

Data integrity measures included IP-based submission limits, built-in logical checks, required response time for core items, and attention-check (reverse-coded) items. All retained data were exported directly from Sojump and cross-checked by 2 independent researchers (RX and HW) to verify data completeness.

### Ethical Considerations

This study received ethics approval by the Ethics Review Committee of the National Clinical Medical Research Center, Second Xiangya Hospital, Central South University (LYG20240038) and adhered to the tenets of the Declaration of Helsinki and its subsequent revisions. To ensure that participants were fully informed, the first page of the survey provided details on the study objectives, estimated completion time, anonymity, voluntary participation, and data management procedures. Electronic informed consent was obtained from all participants prior to questionnaire submission, ensuring their understanding and agreement to participate.

## Results

### General Characteristics

**Figure 1:**
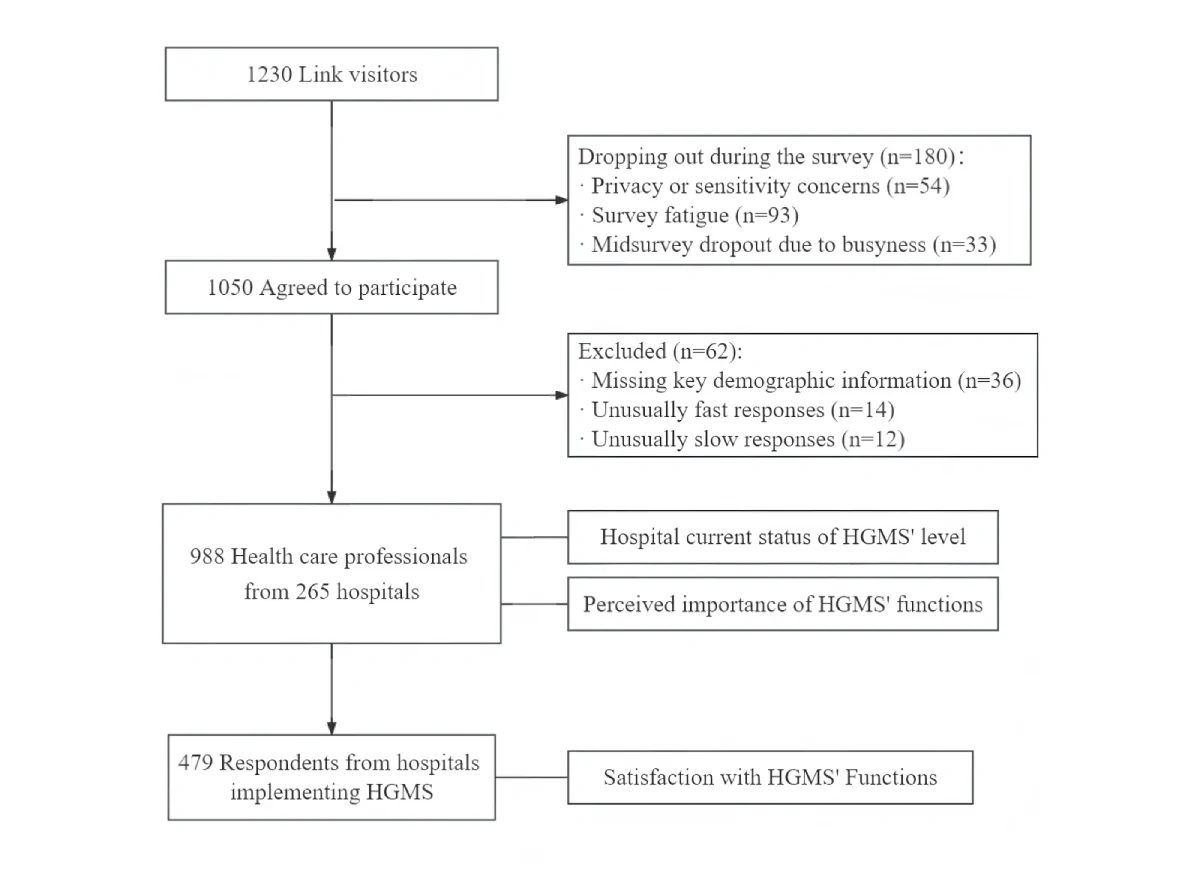
Workflow of the participant selection and survey design. HGMS: hospital-wide glycemic management systems.

A total of 1230 individuals accessed the online survey link, and 1050 provided consent to participate and 988 participants completed the study finally (response rate is 80.33%). During data cleaning, records with missing key demographic information or unusually fast or slow responses were excluded based on relative completion time indices. The final dataset included 988 health care professionals, including 479 with prior experience in glycemic management systems. Figure 1 shows the detailed process.

**Figure 2:**
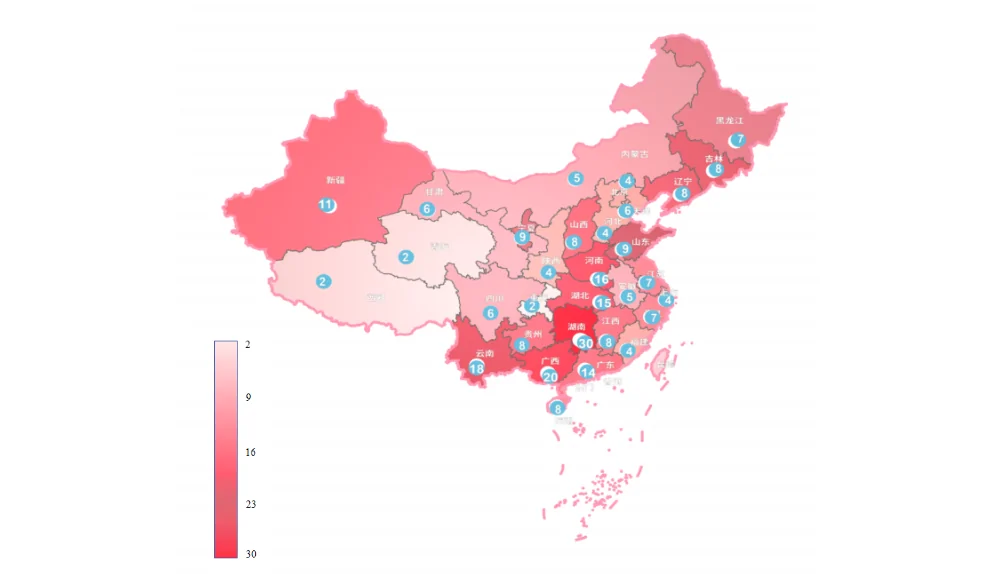
Distribution of participating hospitals.

Participants were recruited from 31 provincial-level administrative regions in the Chinese mainland, ensuring a balanced and representative distribution. The respondents came from 265 hospitals, including 210 (79.25%) tertiary hospitals, 42 (15.85%) secondary hospitals, 6 (2.26%) primary hospitals, and 7 (2.64%) community hospitals. Figure 2 illustrates the geographical distribution of the participating hospitals.

Among the respondents, 90.99% (899/988) were female, and the mean age was 40.79 (SD 4.87) years. Physicians accounted for 22.87% (226/988) of participants, whereas nurses comprised 77.13% (762/988). Most participants (900/988, 91.09%) held a bachelor’s degree or higher. More than half of the participants (572/988, 57.89%) had more than 10 years of work experience. Regarding departmental distribution, most participants worked in internal medicine (661/988, 66.90%), including endocrinology (468/988, 47.37%) and nonendocrinology internal medicine departments (193/988, 19.53%). Detailed participant characteristics are shown in Table 2.

**Table 2:** Characteristics of the participants (N=988).

Characteristics				Respondents
**Sex** **, n (%)**				
		Female	899 (90.99)	
		Male	89 (9.01)	
**Age (years)** **, n (%)**				
		18-29	179 (18.12)	
		30-44	701 (70.95)	
		45-59	108 (10.93)	
**Occupation** **, n (%)**				
		Doctor	226 (22.87)	
		Nurse	762 (77.13)	
**Education** **, n (%)**				
		Junior college or below	88 (8.91)	
		Bachelor’s degree	754 (76.32)	
		Master’s degree or above	146 (14.78)	
**Years of work experience** **, n (%)**				
		＜5	80 (8.10)	
		5-10	336 (34.01)	
		11-20	438 (44.33)	
		＞20	134 (13.56)	
**Professional title** **, n (%)**				
	Junior			242 (24.49)
	Intermediate			553 (55.97)
	Associate senior			135 (13.66)
	Senior			58 (5.87)
**Administrative position** **, n (%)**				
		None	819 (82.89)	
		Yes	169 (17.11)	
**Hospital level** **, n (%)**				
		Tertiary hospital	210 (79.25)	
		Secondary hospital	42 (15.85)	
		Primary hospital	6 (2.26)	
		Community hospital	7 (2.64)	
**Department** **, n (%)**				
		Internal medicine (endocrinology)	468 (47.37)	
		Internal medicine (nonendocrinology)	193 (19.53)	
		Surgery	165 (16.70)	
		Gynecology	19 (1.92)	
		Pediatrics	26 (2.63)	
		Critical care medicine	38 (3.85)	
		Otorhinolaryngology	10 (1.01)	
		Operating department	6 (0.61)	
		Psychiatry and psychology	37 (3.74)	
		Geriatrics and rehabilitation or traditional Chinese medicine	14 (1.42)	
		Administration	12 (1.21)	

### Current Status of HGMS in Chinese Hospitals

**Figure 3:**
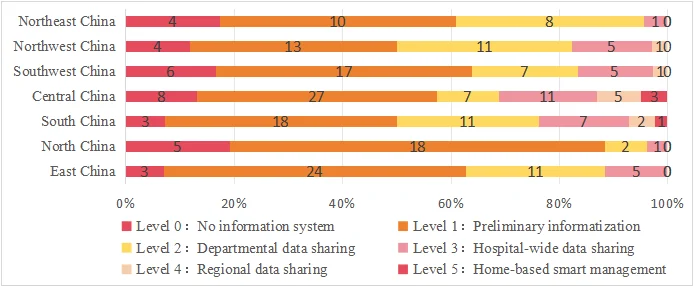
Current status of glycemic management information systems in hospitals across 7 regions of the Chinese mainland.

This study systematically analyzed the application of HGMS in 265 hospitals (Figure 3). The results showed that most hospitals remained in the stages of digitalized glycemic management. Specifically, 47.92% (127/265) were classified as level 1, 21.51% (57/265) at level 2, and 13.21% (35/265) at level 3, indicating that over 80% of hospitals had achieved only preliminary or departmental levels of data sharing. Only a small proportion had reached higher levels, with 3.40% (9/265) at level 4 and 1.51% (4/265) at level 5. Additionally, 12.45% (33/265) of hospitals remained at level 0, indicating an absence of systematic digital glycemic management.

### Assessment of Health Care Professionals’ Perceptions of the Importance and Satisfaction With HGMS

**Figure 4:**
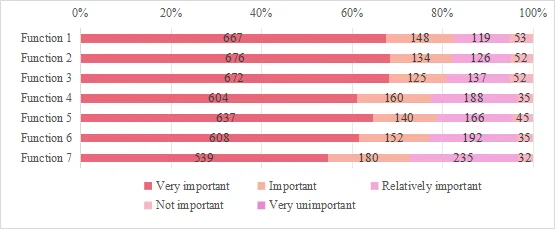
Importance ratings of functions in hospital-wide glycemic management systems.

Health care professionals demonstrated a strong consensus regarding the importance of HGMS functions. The highest proportions of “very important” ratings were reported for function 2 (676/988, 68.42%), function 3 (672/988, 68.02%), and function 1 (667/988, 67.51%) in Figure 4. The Friedman test indicated significant differences in perceived importance across the 7 functions (*χ*²_6_=89.895; *P*<.001). Post hoc Wilcoxon signed-rank tests with Bonferroni adjustment further showed that the relative importance ranked from highest to lowest as follows: (function 1, function 2, function 3, and function 5) > (function 4 and function 6) > function 7 in Table 3.

**Figure 5:**
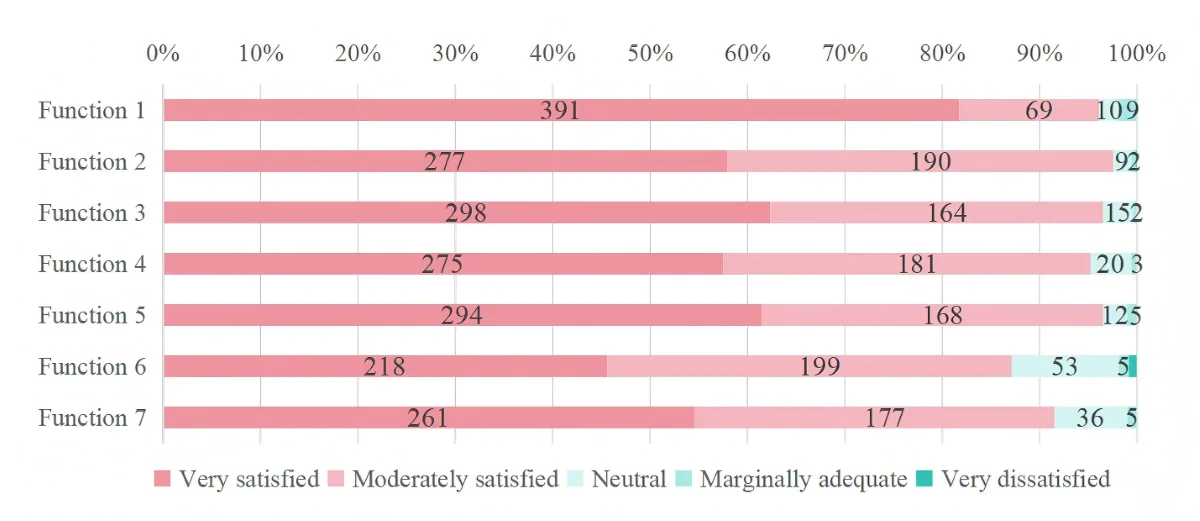
Satisfaction ratings of functions in hospital-wide glycemic management systems.

A satisfaction survey was conducted among 479 health care professionals working in hospitals that had implemented HGMS. Results indicated varying levels of satisfaction across functional modes (Figure 5). Among basic functions, the highest proportion of “very satisfied” responses was observed for function 1 (391/479, 81.63%). Regarding advanced functions, satisfaction was highest for function 5 (294/479, 61.38%), while function 6 received the lowest satisfaction score (218/479, 45.51%). The Friedman test demonstrated significant differences in satisfaction ratings across the 7 functions (*χ*²_6_=286.680; *P*<.001). Post hoc Wilcoxon signed-rank tests with Bonferroni correction indicated that satisfaction ranked from highest to lowest as follows: function 1 > (functions 2, 3, 4, and 5) > function 7 > function 6 in Table 3.

**Table 3:** Friedman test and Wilcoxon signed-rank post hoc comparisons of importance and satisfaction ratings for hospital-wide glycemic management systems functions.

Functions	Importance^a^, mean (SD)	Satisfaction^b^, mean (SD)
1. Automated glucose data transmission	4.41 (0.937)	4.76 (0.582)
2. Real-time hypo- or hyperglycemia alerts	4.44 (0.914)	4.54 (0.580)
3. CPOE-EMR^c^ interoperability	4.44 (0.917)	4.58 (0.576)
4. Tele-endocrinology consultation	4.38 (0.885)	4.52 (0.609)
5. Standardized education and training	4.41 (0.894)	4.57 (0.599)
6. Clinical decision support	4.39 (0.878)	4.30 (0.771)
7. Multidisciplinary remote glucose management	4.29 (0.889)	4.45 (0.679)

^a^*χ*^2^_6_=89.85; *P*<.001. Function 1, function 2, function 3, and function 5 > Function 4 and function 6 > Function 7 on the Wilcoxon signed-rank post hoc test.

^b^*χ*^2^_6_=286.680; *P*<.001. Function 1 > Functions 2, 3, 4, and 5 > Function 7 > Function 6 on the Wilcoxon signed-rank post hoc test.

^c^CPOE-EMR: Computerized Physician Order Entry–Electronic Medical Record.

### Main Themes

We conducted semistructured interviews with 15 professionals based on key quantitative findings: the lowest satisfaction with clinical decision support, low satisfaction with information integration and interdisciplinary collaboration functions, and the general lack of advanced functions such as risk stratification management. The interviews explored underlying reasons, practical challenges, current gaps, and participants’ needs.

The mean age of the participants was 37.13 years, and 11 (73.33%) were female. The mean duration of clinical experience was 11.40 years. Table 4 shows characteristics of the interviewees. All had prior experience in managing patients with abnormal glucose levels as well as in using the HGMS. Subsequent thematic analysis identified three major themes that captured nonendocrinology clinicians’ needs in inpatient glycemic management. Each theme included two dimensions: (1) primary barriers encountered in clinical practice and (2) corresponding functional requirements for intelligent systems (Table 5).

**Table 4:** Characteristics of the interviewees.

Number	Sex	Age (years)	Department	Occupation	Years of work experience	Professional title
1	Female	31	Vascular surgery	Nurse	9	Intermediate
2	Male	36	Endocrinology	Doctor	8	Intermediate
3	Female	42	Endocrinology	Doctor	20	Intermediate
4	Female	39	Nephrology	Nurse	17	Senior
5	Female	37	Ophthalmology	Nurse	15	Senior
6	Female	45	Neurology	Doctor	16	Senior
7	Female	36	Obstetrics	Doctor	2	Junior
8	Female	35	Endocrinology	Doctor	6	Intermediate
9	Female	33	Endocrinology	Doctor	3	Junior
10	Male	31	Orthopedics	Doctor	1	Junior
11	Female	39	Nephrology	Nurse	18	Intermediate
12	Male	44	Liver transplantation	Doctor	16	Senior
13	Female	36	Cardiac surgery	Nurse	14	Intermediate
14	Female	43	General surgery	Nurse	24	Intermediate
15	Male	30	Endocrinology	Doctor	2	Junior

**Table 5:** Summary of qualitative interview themes.

	Insufficient information integration and collaboration	Absence of risk stratification and misaligned management	Insufficient knowledge and education need
Challenges in glycemic management by nonendocrinology clinicians	Incomplete consultation records and delayed cross-departmental communication	Absence of risk-tiered mechanisms in conventional glucose management	Limited proficiency in specialized glycemic management knowledge
Functional requirements	Develop a multisource heterogeneous data integration platform with one-click ward-round functionality	Align glycemic management strategies with patient-specific risk levels	Develop multidimensional training modules encompassing core knowledge, skills training, patient education, and a graded case repository

### Theme 1: Insufficient Information Integration and Collaboration

#### Poor Timeliness in Cross-Departmental Communication

Participants consistently emphasized that effective communication is essential for cross-departmental collaboration. However, current information exchange modalities, mainly mobile messaging and desktop-based systems, were regarded as inefficient due to frequent message delays, missed responses, and slow feedback, all of which hindered coordinated care.

Communication is a major barrier in cross-departmental management. I often need to review medical records to understand the patient’s condition, but it is difficult to access relevant information from other departments at once. The internal messaging system only allows communication within a department, so I often have to use external platforms such as WeChat to contact them.

Participant 1

#### Functional Requirements for Developing a Multisource Heterogeneous Data Integration Platform

Participants indicated that HGMS facilitate seamless communication and data sharing through an integrated, high-performance interface. This platform should provide real-time access to multisource patient data from hospital information system, laboratory information system, and glucose-monitoring systems. It should automatically extract and correlate key parameters such as medication records, nutrition data, and glucose trends to support efficient visualization and clinical decision-making.

It would be ideal if the system included a dedicated communication function that allows direct contact with the glycemic management team and timely expert feedback. The system should also present a clear overview of the patient’s condition, including glucose values, dietary records, and physical activity.

Participant 7

The efficiency of relaying endocrinology recommendations is low. The system should support direct communication and blood glucose data sharing, minimizing the need for in-person consultations.

Participant 12

### Theme 2: Absence of Risk Stratification and Misaligned Management

#### Lack of Risk-Tiered Mechanisms in Conventional Blood Glucose Management

Conventional management approaches lack a systematic framework for risk stratification and therefore focus on short-term stabilization rather than sustained, risk-based care. Clinical interventions primarily address acute glycemic fluctuations, while risk-stratified, targeted management strategies remain underdeveloped. In addition, endocrinologist involvement is often limited to single consultation without structured mechanism for continuous collaboration. Consequently, deficiencies in risk identification and resource allocation significantly constrain the effectiveness of glycemic management.

We frequently focus on short-term outcomes. When patients present with hyperglycemia, insulin is administered once for temporary control, without consideration for sustained glycemic stability or long-term management planning.

Participant 14

The consultation process is excessively time-consuming. The endocrinology team usually conducts only a single assessment, and any subsequent adjustments require repeated consultation, which is inefficient.

Participant 5

#### Functional Requirements for Aligning Glycemic Management Strategies With Patient-Specific Risk Levels

Participants emphasized that the HGMS should enable automated patient stratification at enrollment, with high-risk features flagged immediately and individualized glycemic targets pushed to the relevant departmental terminals. Establishing standardized, hospital-wide stratification protocols that integrate key indicators such as etiology and treatment regimen was considered essential for optimizing specialist resource allocation.

We should classify patients hospital-wide based on their disease profiles. For example, orthopedic patients on steroids need close postoperative glucose monitoring, gastrointestinal patients require a focus on nutritional support, and patients with hepatic or renal dysfunction should receive tailored dietary guidance. Department-specific management would be far more effective.

Participant 15

Participants further highlighted the need for a dynamic, risk-tiered response mechanism that adjusts the intensity of specialist involvement according to case complexity. By automatically analyzing clinical characteristics and matching patients with appropriately qualified health care teams, the system could promote efficient resource usage and ensure precision care across all risk levels.

Poor glycemic control in nephrology patients accelerates renal deterioration and may lead to end-stage kidney disease. If the system could automatically identify and flag such high-risk cases, endocrinology could prioritize them for senior physicians.

Participant 4

Some participants advocated integrating context-specific pathways, treatment recommendations, and safety alerts, while others cautioned against overreliance on automated decision support.

If the system could provide emergency management for hypo-/hyperglycemia or suggest medication adjustments when glycemic targets are not met, it would significantly reduce manual workload.

Participant 11

The system should generate intelligent alerts for antidiabetic drug selection based on liver and kidney function. For example, it should warn if a prescribed oral agent is contraindicated or prompt medication adjustment when renal function declines. However, we should also recognize that automated alerts can just serve as auxiliary tools. Clinical decision-making must still be guided by physicians’ professional judgment to avoid overreliance on system recommendations.

Participant 3

### Theme 3: Insufficient Knowledge and Education Needs

#### Limited Proficiency in Specialized Glycemic Management Knowledge

Endocrinologists reported that nonspecialist physicians often demonstrated limited understanding of glycemic targets and insufficient familiarity with evidence-based antihyperglycemic management. Knowledge gaps were particularly evident for insulin administration protocols and for the appropriate use of oral agents. These deficiencies frequently led to delayed or inadequate responses to dysglycemia, resulting in suboptimal glucose control and increased risk of acute diabetes-related complications.

Their understanding of blood glucose control often falls into two extremes. Some have little awareness of age-specific glycemic targets and become overly anxious when glucose is only slightly elevated. Others fail to recognize problems until glucose levels become extremely high, sometimes exceeding 20 mmol/L.

Participant 9

Surgeons generally have limited knowledge of glucose management, and some are unfamiliar with the types of insulin. Internists tend to have a better grasp of oral hypoglycemics, yet their understanding remains incomplete, particularly regarding adverse effects. For example, some cardiologists may prescribe both GLP-1 receptor agonists and SGLT-2 inhibitors but fail to adjust treatment even when gastrointestinal symptoms occur.

Participant 8

Nonendocrinology health care providers also commonly lacked the expertise needed to deliver effective diabetes education. Physicians without diabetes care may struggle to help patients in practical self-management, and nurses without standardized training may be unable to provide structured health education. Together, these limitations weaken the overall capacity for patient self-management.

Patient education is crucial because 90% of diabetes management relies on self-care. Poor post-discharge glycemic control is common, and complications such as diabetic ketoacidosis often result from long-term deficiencies in self-management. While medical intervention is essential, strengthening patients’ ability to manage their own condition is the key to improving glycemic control.

Participant 2

Our own knowledge is insufficient to fully implement the comprehensive five-horse carriage model, especially when developing individualized dietary plans. We can only provide general guidance, such as suggesting patients to choose low-glycemic-index foods, which does not meet the diverse needs of different individuals.

Participant 6

#### Functional Requirements for Development of Multidimensional Training Modules

Participants emphasize the need for comprehensive and targeted training programs that use multiple instructional formats to enhance knowledge retention and facilitate guideline implementation. Such programs would strengthen providers’ professional competence and improve the quality of patient education. Incorporating direct-to-patient educational tools was also considered essential for creating an effective closed-loop management system.

Training needs to be strengthened across all components of glycemic management. Some physicians are not familiar with the hypoglycemia management process. A comprehensive, systematic training program presented through text, images, and videos would help them better understand key concepts and workflows.

Participant 10

We could offer patients concise bedside handouts with essential reminders, while detailed materials could be accessed via QR codes or pushed directly to their mobile devices.

Participant 13

## Discussion

### Principal Findings

HGMS in hospitals in the Chinese mainland are generally transitioning from basic electronic record-keeping to information-based management systems, and its application remains largely concentrated in endocrinology departments. Core functions such as real-time alerts and automated data transfer are widely recognized. In contrast, advanced functions, including clinical decision support, multidisciplinary remote glucose management, and telemedicine, showed lower levels of acceptance and satisfaction. Integration of the quantitative and qualitative findings suggests that these gaps may be attributable to persistent barriers in interdepartmental information sharing, limited risk-stratified management pathways, insufficient decision-making support for nonspecialist clinicians, and unmet training needs. These findings suggest that optimizing glycemic management requires technological advancement alongside organizational collaboration, workflow redesign, and capacity building, with future strategies focusing on strengthening foundational functions while integrating advanced applications into routine clinical workflows.

### Marked Regional Disparities in HGMS Across the Chinese Mainland

Significant regional disparities exist in the digital maturity digitalization in hospital glucose management across the Chinese mainland, a pattern aligned with findings from Ye et al [23] on regional heterogeneity in health informatics development. In the northern China, such as Inner Mongolia, some hospitals remain at level 0, characterized by limited digital infrastructure and reliance on paper-based or partially electronic workflows. In eastern regions such as Jiangxi Province, most hospitals are at level 1, where data are manually entered into electronic systems, indicating an early stage of digital transition. In Northeast China, including Liaoning, Jilin, and Heilongjiang Provinces, hospitals have generally reached level 2, with fully electronic glucose recording and partial data sharing across departments. In southern regions such as Guangdong and Guangxi, hospitals generally show more advanced digital infrastructures, with several hospitals reaching level 3, characterized by hospital-wide data integration and real-time glucose monitoring [19]. In contrast, in Southwest China, such as Sichuan Province, several high-tier hospitals have reached level 4, enabling regional data sharing [23]. Building on these developments, hospitals in Central China, such as Hunan and Hubei, have progressed to level 5, achieving home-based smart glucose management through data-transmitting glucometers. Patients use data-transmitting glucometers at home, enabling physicians to conduct real-time remote monitoring and provide timely clinical recommendations. This model aligns with the collaborative health care networks described by Rushakoff and colleagues [24], supporting intelligent and regionally integrated diabetes management systems. However, tertiary hospitals were overrepresented in the sample (79.25%), while primary and community health care institutions accounted for less than 5%. As tertiary hospitals typically have more advanced IT infrastructure and resources, the findings may overestimate national HGMS implementation and underestimate the proportion of hospitals at levels 0-1.

In contrast, hospitals in many high-income countries are generally at level 3 or higher. Many hospitals in the United States and Europe have reached level 3, implementing hospital-wide glucose data integration, real-time visibility of glucose values, and alert-based clinical decision support within electronic medical records [25]. Some tertiary centers have advanced to level 4, characterized by regional data sharing and coordinated management across affiliated hospitals. These centers use centralized review of inpatient glucose data and specialist recommendations [26], along with daily inpatient glycemic survey systems [27], to support cross-hospital evaluation and facilitate timely clinical interventions. A subset of advanced centers has reached level 5 through the implementation of home-based smart glucose management systems, enabling remote monitoring and clinical recommendations. These systems demonstrate the feasibility of virtual and patient-centered models of diabetes care [28].

Overall, hospitals in the Chinese mainland have made substantial progress in digital glycemic monitoring. However, system interoperability, standardized clinical decision support, and predictive analytics remain underdeveloped. The Chinese mainland has established foundational digital capabilities glycemic management. Further adoption of integrated clinical decision support, virtual management, and remote monitoring strategies could enhance hospital-wide glycemic monitoring, improve cross-department data visibility, and support timely clinical decision-making.

### Challenges and Needs of Nonspecialist Health Care Teams in Glycemic Management

This study compared endocrinology specialty wards with nonspecialty wards and identified multidimensional disparities in the digitalization of glycemic management processes. Endocrinology wards have established a comprehensive glycemic management system, encompassing continuous monitoring and individualized interventions [28]. In contrast, nonspecialty wards continue to emphasize basic data collection and documentation rather than structured glycemic management. From a management perspective, endocrinology wards adopt guideline-driven approaches supported by multidisciplinary teams, enabling precision glycemic management strategies consistent with the model proposed by Samson and colleagues [29]. Conversely, nonspecialty wards exhibit fragmented management practices, often marked by delayed consultation responses and inconsistent implementation of interventions [18]. The quality of glycemic management in nonspecialty wards is influenced by multiple factors, including inefficiencies in consultation and collaboration mechanisms, limited knowledge required for glycemic decision-making, and disparities in professional competence [30]. This qualitative finding is consistent with the quantitative result showing that multidisciplinary remote glycemic management (function 7) received the lowest importance rating (mean 4.29, SD 0.889), suggesting that ineffective interdepartmental communication practices may diminish health care professionals’ recognition of the value of this function. Specifically, consultation delays and fragmented processes contributed to a reactive rather than proactive approach to abnormal glycemic events [31]. Limited access to endocrinology support often compelled nonspecialist clinicians to make complex clinical decisions despite restricted expertise [16]. For patients with glucose fluctuations or comorbidities, nonspecialist physicians frequently lacked adequate evidence-based knowledge to set appropriate glycemic targets. They also demonstrated limited familiarity with the indications, contraindications, and clinical applicability of antidiabetic agents [32]. This qualitative finding is also consistent with the quantitative result that clinical decision support (function 6) had the lowest satisfaction rate (50.4%), indicating that nonspecialist physicians’ knowledge gaps in medication-related decision-making may require systematic decision support tools to facilitate evidence-based practice. Additionally, the quality of self-management education delivered by nursing teams varied considerably, falling to meet the differentiated needs of patients across risk categories [33]. These findings align with observations of Byrne et al [34], who emphasized the necessity of enhanced training for nonspecialist health care providers in glucose management. This need is further supported by the quantitative findings, where standardized education and training were rated as highly important (mean 4.41, SD 0.894), yet achieved a satisfaction rate of only 63.5%, indicating opportunities for further improvement.

### Future Directions for Integrated and Intelligent Hospital-Wide Glycemic Management

The study identified significant discrepancies between health care professionals’ perceived importance and satisfaction of functions within HGMS. Foundational functions received the higher ratings in both perceived importance and user satisfaction, reflecting a core clinical need for standardized and automated glucose management workflows. However, this alignment diminished for advanced functions, which exhibited a pronounced importance-satisfaction mismatch, revealing the critical functional gap in the current digital advancement of HGMS.

**Figure 6:**
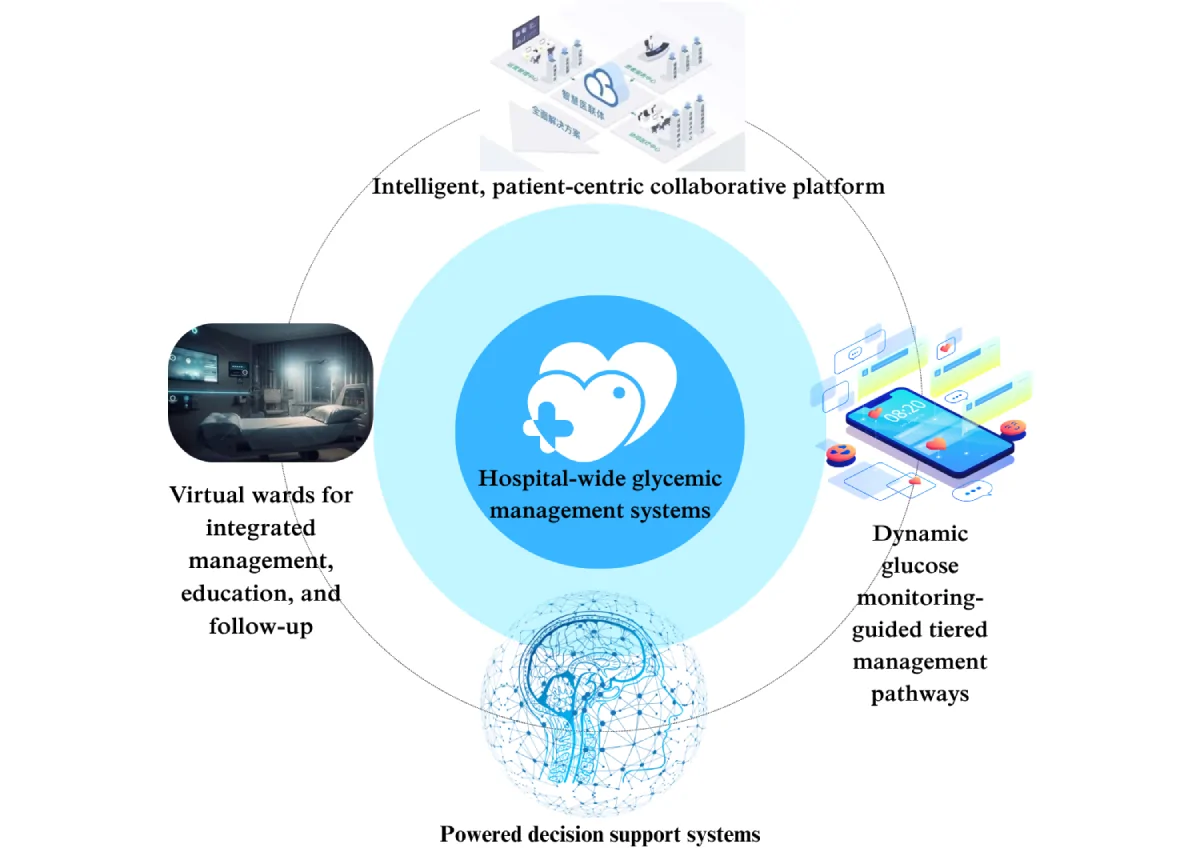
Future development directions of hospital-wide glycemic management systems.

This gap is further reflected in the glycemic management challenges of nonspecialist wards, which are driven by 3 main factors: poor information integration and collaboration, absence of risk stratification with misaligned care pathways, and inadequate clinician training. Addressing these gaps requires multidimensional interventions, including optimization of clinical pathways, improved information systems and interoperability, and targeted allocation of endocrinology expertise and related support resources. Accordingly, we propose four key directions for system optimization [35,36] (Figure 6): (1) establishing virtual wards for integrated management, education, and follow-up; (2) developing dynamic, glucose-monitoring–guided tiered management pathways; (3) building an intelligent, patient-centered collaborative platform; and (4) deploying interpretable AI-assisted clinical decision support to augment clinician judgment. These approaches are mutually reinforcing and collectively promote the development of an integrated and intelligent HGMS.

The virtual wards could serve as the foundational infrastructure for integrated management, education, and follow-up. It would enable real-time data collection and apply intelligent alert algorithms to identify patients who require intervention, with automatic notifications directed to endocrinology teams. Alert algorithms must be validated for clinical performance and tuned to minimize false positives and alert fatigue [37]. The system should include virtual wards’ function, allowing specialists to remotely monitor glycemic trends and provide individualized recommendations [38]. In addition, dynamic tiered management pathways guided by glucose monitoring data should be established. These pathways would integrate continuous glucose monitoring data and point-of-care glucose testing to generate evidence-based risk prediction models. Such models would support a shift from reactive management to proactive, risk-stratified prevention [15]. To reduce collaboration barriers, an intelligent, patient-centric platform should consolidate clinical data and consultation workflows into a unified interface. This platform should support interoperability standards, secure role-based access, and structured handoffs to enable coordinated workflows among endocrinology, nutrition, pharmacy, and primary care teams [17]. Core functions should include automated task allocation based on patient risk pathways, structured documentation templates, and closed-loop tracking of recommendations to ensure timely implementation and feedback [39]. AI-assisted decision support systems should prioritize interpretable models aligned with evidence-based guidelines so that clinicians can understand the rationale for recommendations [40]. Human oversight must be maintained, with clinicians retaining final decision authority to prevent overreliance. Patient-facing outputs should use clear visualizations of the decision logic to enhance understanding and support trust and informed consent [41]. Overall, these strategies will support a safe, precise, and continuous intelligent glycemic management system that enhances hospital-wide care and contributes to digital transformation in chronic disease management.

### Limitations

This exploratory study provides a cross-sectional overview of the informatization of glycemic management in the Chinese mainland but does not capture its longitudinal evolution or long-term clinical effects. The overrepresentation of tertiary hospitals in the sample may limit the generalizability of the findings to primary care settings. Data collection through self-reported questionnaires and interviews may introduce response bias, particularly when evaluating satisfaction with system functionalities. Although thematic saturation ensured analytical depth in the qualitative component, the modest sample size may constrain the transferability of results. In addition, the absence of patient perspectives limited assessment of the impact of information-based management on clinical outcomes, which warrants further investigation.

### Conclusions

This nationwide multicenter survey systematically characterized the informatization of glycemic management across multiple levels in hospitals in the Chinese mainland. Findings suggest that current glycemic management practices are transitioning from electronic documentation to intelligent management. Core functionalities, such as automated data uploads and real-time alerts, were highly recognized, whereas more advanced functions, including clinical decision support and multidisciplinary remote glucose management, were variably adopted. Furthermore, regional disparities were evident. These results provide empirical evidence into the progress and uneven development of medical informatization in the Chinese mainland. The qualitative analysis further revealed critical challenges in nonspecialty settings, including inefficient cross-department collaboration, absence of standardized patient stratification, and insufficient professional training. Future improvements should focus on four directions: (1) building specialized virtual wards to facilitate hospital-wide access to endocrinology expertise, (2) establishing dynamic monitoring and stratified systems to enhance precision in glycemic management, (3) developing multidisciplinary collaboration platforms to streamline cross-professional workflows, and (4) deploying interpretable AI-assisted decision support systems that preserve clinical autonomy while improving efficiency. Together, these strategies may facilitate the transition of glycemic management from fragmented practices to standardized protocols and from reactive response to proactive prevention.

## Abbreviations

HGMS: hospital-wide glycemic management systems

## Data Availability

The data that support the findings of this study are not publicly available due to privacy and ethical restrictions but are available from the corresponding author upon reasonable request.
